# Treatment of Tuberculous Glands

**Published:** 1893-01-21

**Authors:** 


### BRISTOL GENERAL HOSPITAL.

### TREATMENT OF TUBERCULOUS GLANDS.

Mr. Penny in the early stage lays great stress on the improvement of general hygiene conditions—fresh air, tonics, preparations of iron in the form either of the compound syrup of the phosphate of iron or the syrup of iodide of iron, cod liver oil, and port wine. He also uses the ointment of iodide cadimum or oleate of mercury, and iodine applications. When the glands have begun to soften and suppurate, he opens the softening mass and scrapes out the caseous material freely, until firm tissue is reached, and then applies to the cavity a solution of chloride of zinc of the strength of forty grains to the ounce of water. If the abscess has already opened he slits up the undermined skin, and scrapes round under it, and trims off any overhanging bits. Small holes in the facia are to be carefully looked for. They often lead into further suppurating cavities beneath, and when practicable must be carefully laid open, and the caseous matter beneath as carefully scraped away as in the more superfical collections. Volkman's spoon is the best instrument to remove the caseous tubercular tissue with. If there should be free oozing a piece of "protective" is placed next the wound and into cavity, and the part plugged. The "protective" will enable it all to slip away the next day without disturbing the clots. The dressing consists of boracic lint, or boracic or iodoform ointment, and if there is much discharge, a layer of wood wool wadding.

